# Bidirectional Echolocation in the Bat *Barbastella barbastellus*: Different Signals of Low Source Level Are Emitted Upward through the Nose and Downward through the Mouth

**DOI:** 10.1371/journal.pone.0135590

**Published:** 2015-09-09

**Authors:** Anna-Maria Seibert, Jens C. Koblitz, Annette Denzinger, Hans-Ulrich Schnitzler

**Affiliations:** Animal Physiology, Institute for Neurobiology, University of Tübingen, Auf der Morgenstelle 28, 72076 Tübingen, Germany; Lund University, SWEDEN

## Abstract

The Barbastelle bat (*Barbastella barbastellus*) preys almost exclusively on tympanate moths. While foraging, this species alternates between two different signal types. We investigated whether these signals differ in emission direction or source level (SL) as assumed from earlier single microphone recordings. We used two different settings of a 16-microphone array to determine SL and sonar beam direction at various locations in the field. Both types of search signals had low SLs (81 and 82 dB SPL rms re 1 m) as compared to other aerial-hawking bats. These two signal types were emitted in different directions; type 1 signals were directed downward and type 2 signals upward. The angle between beam directions was approximately 70°. Barbastelle bats are able to emit signals through both the mouth and the nostrils. As mouth and nostrils are roughly perpendicular to each other, we conclude that type 1 signals are emitted through the mouth while type 2 signals and approach signals are emitted through the nose. We hypothesize that the “stealth” echolocation system of *B*. *barbastellus* is bifunctional. The more upward directed nose signals may be mainly used for search and localization of prey. Their low SL prevents an early detection by eared moths but comes at the expense of a strongly reduced detection range for the environment below the bat. The more downward directed mouth signals may have evolved to compensate for this disadvantage and may be mainly used for spatial orientation. We suggest that the possibly bifunctional echolocation system of *B*. *barbastellus* has been adapted to the selective foraging of eared moths and is an excellent example of a sophisticated sensory arms race between predator and prey.

## Introduction

Barbastelle bats (*Barbastella barbastellus*) are one of the most specialized Palaearctic bats [1], preying almost exclusively on lepidoptera (up to 99% by volume) [1,2,3], and preying particularly on small tympanate moths [4]. Barbastelles belong to the guild of edge space aerial-hawking foragers [5,6] and feed mainly above the canopy [1] but also in woodlands, open grasslands, and rocky landscapes [7,8,9].

The echolocation behavior of *B*. *barbastellus* is unique among the European Vespertilionids. Barbastelle bats emit two different search call types [1,3,10,11,12,13,14], designated as type 1 and type 2 calls [15]. The more stereotyped type 1 FM-signals are shorter and sweep from 36–28 kHz, while type 2 signals are longer and cover a frequency range of 45–32 kHz. Research using single microphone recordings suggested that the two call types of *B*. *barbastellus* varied in amplitude or in emission direction, with type 1 calls having higher amplitudes than type 2 calls [1,10,14,15]. Whether this variation in amplitude is caused by a change in emission direction by vertical head movements [15], or by utilizing a deliberate change of source level (SL), could not be determined. Additionally, different emitters could be involved with the two signal types, as bats have been observed taking off and emitting type 2 echolocation signals with their mouth shut [16,17]. When either the nose or mouth was experimentally plugged, *B*. *barbastellus* could still fly, orientate, and emit signals with similar oscillograms. Only when both the mouth and nostrils were plugged did the bats fail to orientate during flight [17].

Several hypotheses have been suggested to explain the function of these two alternating signals. The more narrowband low-frequency type 1 signal could be better suited for detection, whereas the higher and more broadband type 2 signal could be better suited for the exact localization of targets [14,15]. Recent research has found evidence to suggest that barbastelle bats use signals that are 10–100 times weaker than those of other aerial hawking bats to prevent early detection and evasive behavior by moths. While moth neurons always react to loud search signals emitted by *Nyctalus leisleri* prior to the bat detecting the moth echoes, these same neurons failed to detect the “stealth echolocation” signals of *B*. *barbastellus* [4].

Here we address whether the alternating signal amplitude previously recorded using single microphones reflects a change in emission direction, or alternatively a change in signal sound pressure level. To accomplish this, we used a large microphone array to record the echolocation signals of barbastelle bats while flying towards the array and emitting both types of search signals. With this method it was possible, for the first time, to determine both the SL and the emission direction of the recorded signals. We also investigated how nose and mouth morphology contributes to emission direction in barbastelle bats.

## Results

### Flight and echolocation behavior

**Fig 1:**
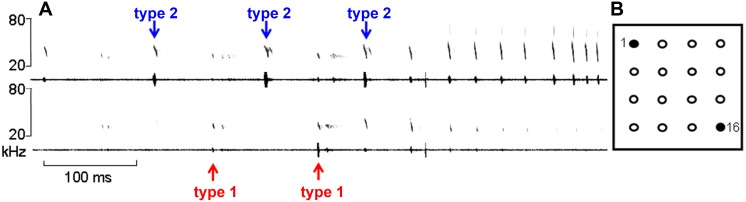
Echolocation signals of *B*. *barbastellus* recorded from different microphone positions. (A) Spectrograms and oscillograms of echolocation signals emitted when flying towards the array (FFT: 256, Hann window). (B) The top left microphone of the array (number 1) recorded the signals in the upper half of (A), whereas the signals in the lower half of (A) were recorded with the low right microphone (number 16). The upper microphone recorded type 2 and approach signals with higher amplitudes, whereas the lower microphone recorded type 1 signals with higher amplitudes.

We recorded barbastelle bats flying towards a microphone array after leaving their roost. The bats emitted type 1 and type 2 signals in an alternating fashion. Type 1 signals always had a higher sound pressure level (SPL) recorded at the lower microphones whereas type 2 signals had higher SPLs at the upper microphones. At a distance of 1–2 m to the array the bats switched to approach calls. During this approach phase they no longer emitted type 1 calls, and transformed type 2 calls into broadband approach calls (Fig 1A and 1B). Bats had an average flight speed of 5.3 m/s and flew 2–6 m above ground.

### Echolocation call parameters

Many of the recorded signals were not centered within the array plane, however, the differences in apparent source level (ASL) and source level (SL) of a call type were minimal with just 1.1–2.5 dB and a SD of 4–7 dB (for details see method section). Therefore we pooled data from all signals of a type to measure signal parameters except for ASL and SL. All measured parameters differed significantly between the two search signal types, except for SL (all results and statistics in Table 1). The mean pulse interval between a type 1 call and a following type 2 call was 67.9 ms and between a type 2 call and its following type 1 call 59.3 ms. Mean call duration of type 1 calls was 1.7 ms, type 2 calls had a mean duration of 2.5 ms. Start frequency of type 1 was 35.9 kHz, that of type 2 44.3 kHz. Type 1 signals ended with a mean terminal frequency of 31.2 kHz, type 2 calls ended at 35.1 kHz. Mean bandwidth in type 1 calls was 4.7 kHz, that of type 2 calls was 9.2 kHz. The peak frequency of type 1 calls measured 33.6 kHz, and for type 2 calls 40.1 kHz. The apparent source level (ASL) of type 1 signals averaged 79.9 dB SPL re 20 μPa rms re 1 m and that of type 2 signals was 82.4 dB SPL rms. Type 1 calls had an absolute SL of 80.9 dB SPL rms re 1 m and type 2 calls had a SL of 82.0 dB SPL rms.

**Table 1:** Signal parameters of type 1 and type 2 echolocation calls.

call type		type 1	type 2	*t*	*p*	significance level
		*N = 24 (n = 86)*	*N = 23 (n = 110)*			
**duration [ms]**	mean (± SD)	1.7 (± 0.4)	2.5 (± 0.4)	*7*.*10*	*< 0*.*0001*	***
	range	1.1–2.4	1.8–3.1			
		*N = 9 (n = 19)*	*N = 7 (n = 43)*			
**pulse interval [ms] to preceeding call**	mean (± SD)	67.9 (± 12.8)	59.3 (± 11.4)			
	range	49.0–92.1	42.3–92.0			
		*N = 24 (n = 86)*	*N = 23 (n = 110)*			
**start freq [kHz]**	mean (± SD)	35.9 (± 1.3)	44.3 (± 0.9)	*25*.*89*	*< 0*.*0001*	***
	range	33.4–38.0	42.4–46.2			
		*N = 24 (n = 86)*	*N = 23 (n = 110)*			
**terminal freq [kHz]**	mean (± SD)	31.2 (± 0.8)	35.1 (± 1.9)	*9*.*19*	*< 0*.*0001*	***
	range	29.6–32.5	31.0–38.4			
		*N = 24 (n = 86)*	*N = 23 (n = 110)*			
**bandwidth [kHz]**	mean (± SD)	4.7 (± 1.0)	9.2 (± 1.9)	*10*.*37*	*< 0*.*0001*	***
	range	3.2–6.4	6.5–12.9			
		*N = 24 (n = 86)*	*N = 23 (n = 110)*			
**peak freq [kHz]**	mean (± SD)	33.6 (± 1.1)	40.1 (± 1.2)	*18*.*99*	*< 0*.*0001*	***
	range	31.7–36.3	37.5–41.7			
		*N = 24 (n = 86)*	*N = 23 (n = 110)*			
**apparent source level [dB]**	mean (± SD)	79.9 (± 5.1)	82.4 (± 3.6)	*1*.*90*	*= 0*.*0642*	**n.s.**
	range	70.0–90.0	76.4–88.1			
		*N = 8 (n = 14)*	*N = 7 (n = 11)*			
**source level [dB]**	mean (± SD)	80.9 (± 7.5)	82.0 (± 4.1)	*0*.*37*	*= 0*.*7206*	**n.s.**
	range	70.0–90.2	77.1–87.8			

Parameters of echolocation signals in *Barbastella barbastellus* emitted when flying towards the microphone array. For each sequence only the mean of the parameters was used so as to avoid pseudoreplication. *N* = number of recorded sequences, *n* = number of signals contained in the sequences used to calculate the corresponding mean.

### Sonar beams and beam direction

**Fig 2:**
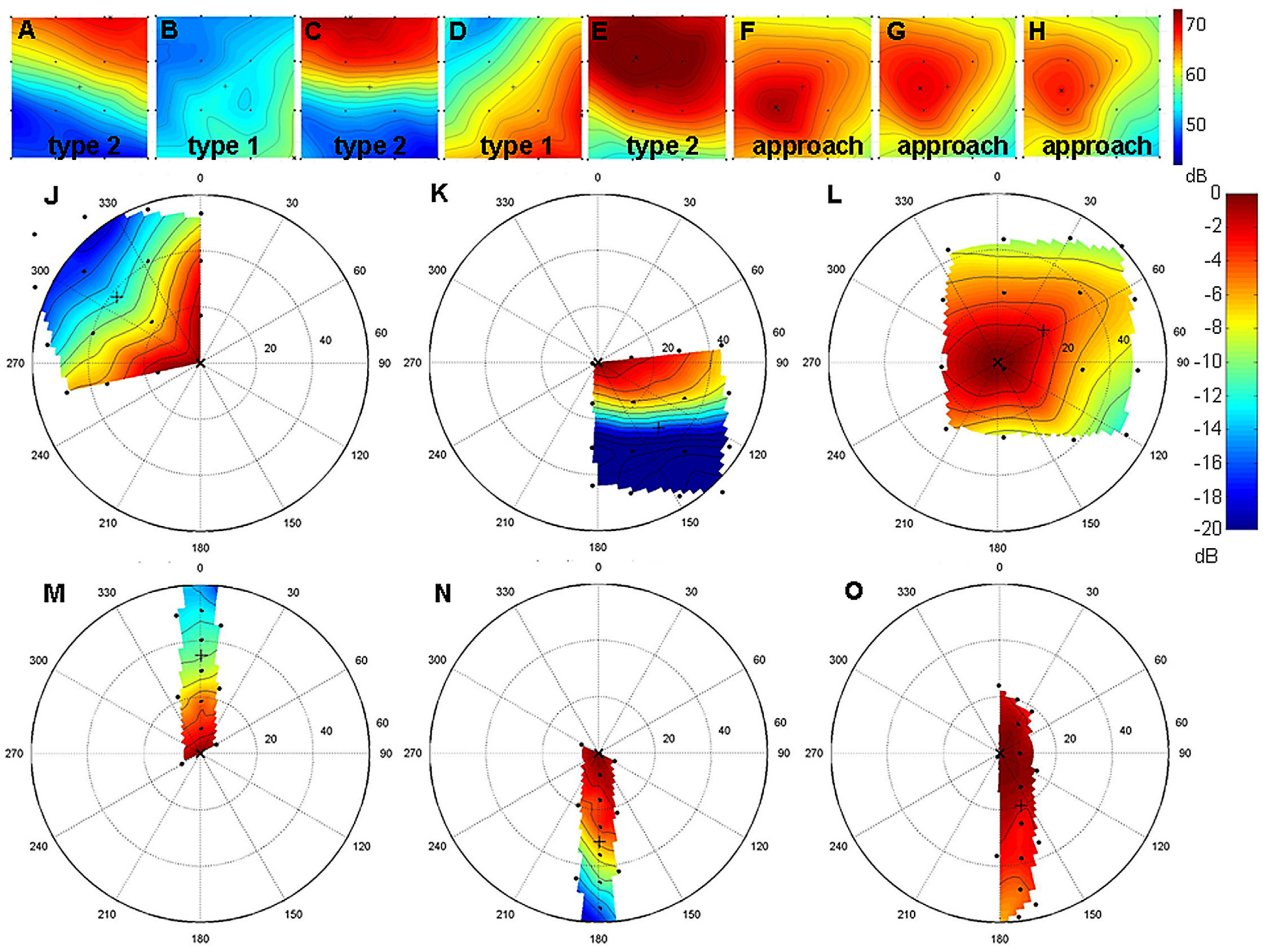
Sonar footprints and reconstructed sonar beams. (A-H) Sonar footprints of type 2 (A,C,E), type 1 (B,D) and approach signals (F-H) of the call sequence depicted in Fig 1 on the square array. The SPL is color-coded and interpolated between the microphones (black dots) on the array plane. The black “+” represents the center of the array, the black “x” the calculated maximum. (J-O) Reconstructed sonar beams of signals recorded with the square array (J-L) and the chain array (M-O). The SPL is color-coded and indicates the beam shape relative to the beam maximum in the center of a polar plot. The black dots mark the positions of the microphones, the black “x” marks the calculated apparent or real beam maximum. Type 1 signals are depicted in (J) and (M), type 2 signals in (K) and (N), and approach signals in (L) and (O). Note that type 1 signals are directed to the lower part of the array whereas type 2 signals are directed to the upper part.

**Fig 3:**
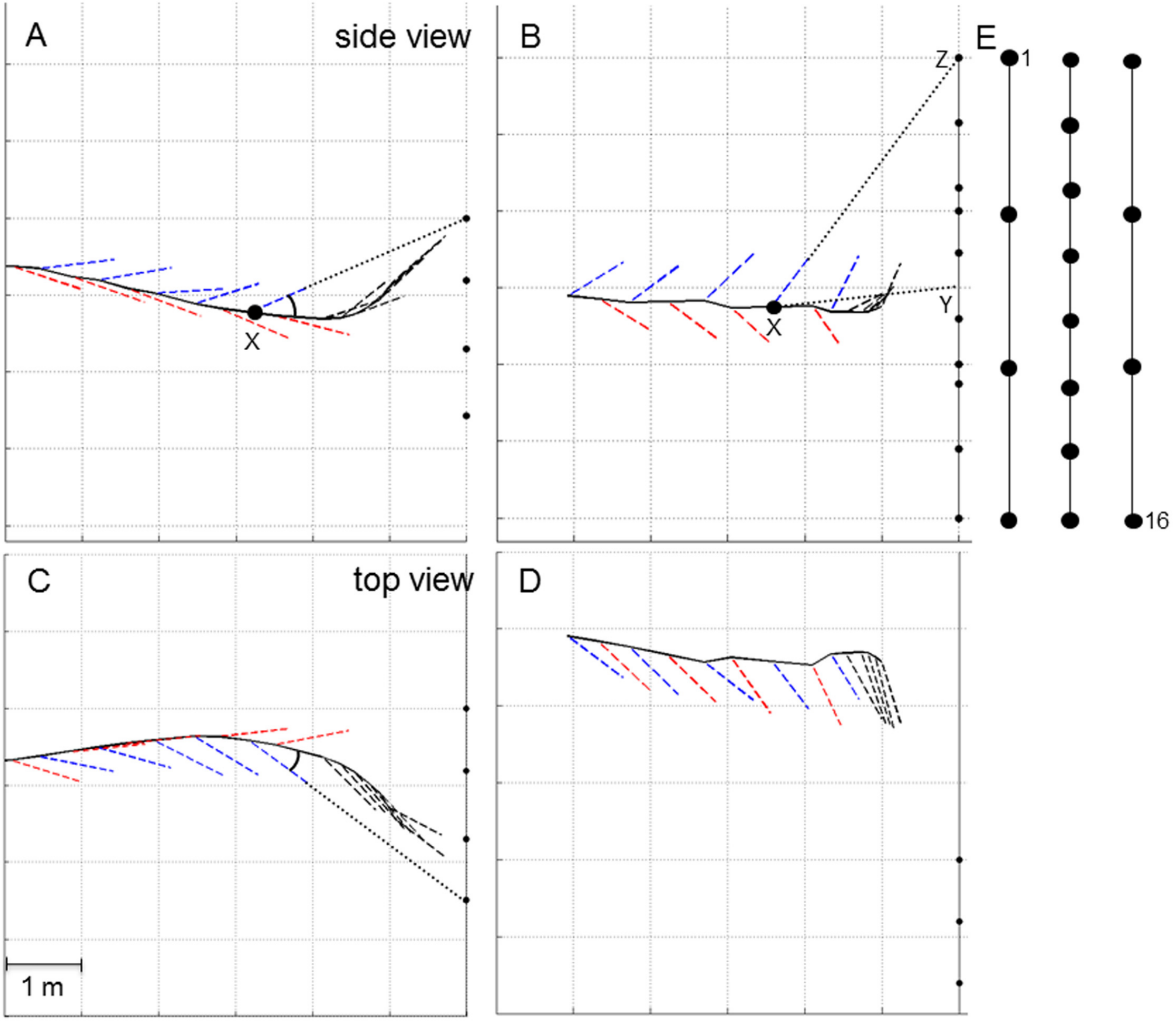
Changes in beam direction in exemplary flights towards the square and the chain array. (A,B) depict side views and (C,D) overhead views of exemplary flights. The black dots represent the microphones in the array. The 4 m high square array was positioned ~1.6 m above ground (A,C) while the 6 m high chain array was positioned about 0.3 m above ground (B,D). The flight paths are depicted as black lines. At each position where a signal was emitted a vector pointing towards the calculated apparent beam maximum on the array indicates the apparent beam direction. The vectors of type 2 signals are depicted in blue and those of type 1 signals in red. Black vectors indicate the direction of approach signals. Note that in the flight depicted in (B,D), the bat passed on the left side of the chain array. Thus all horizontal beam directions are artifacts pointing to the right side whereas the bat might be facing straight ahead. However, the vertical angles remain unaffected by this offset. The vertical and horizontal angles between flight direction and apparent beam direction for each bat position (X) as shown in (A) and (C) are displayed in Fig 4. The vertical angle between the direction from the position of the bat (X) to the center of the microphone array (Y) and the apparent beam direction to the upper (Z) or lower end of the array as shown in (B) is displayed in Fig 5. (E) Front view scheme of the chain array with the first (1) and last microphone (16) labeled.

The reconstructed sonar beams showed a clear pattern of alternating directions. Type 1 signals usually had its apparent beam maximum on the lower part of the array, while type 2 calls showed apparent beam maxima on the upper array edges (Fig 2J and 2K). This pattern was also found in the recordings of the 6 m high chain array (Fig 2M and 2N). The fact that approach signals also pointed upward suggests that these signals are derived from type 2 signals. During approach, bats focused on the array resulting in beam maxima within the array (Fig 2L and 2O). A complete sequence of alternating signals and three approach signals is depicted in Fig 2A–2H. Vectors pointing from the bat to the apparent or real beam maximum indicate beam direction (Fig 3). These reconstructions, too, showed the distinct alternating pattern of type 1 signals pointing downward and type 2 signals pointing upward.

### Apparent angles between call types

**Fig 4:**
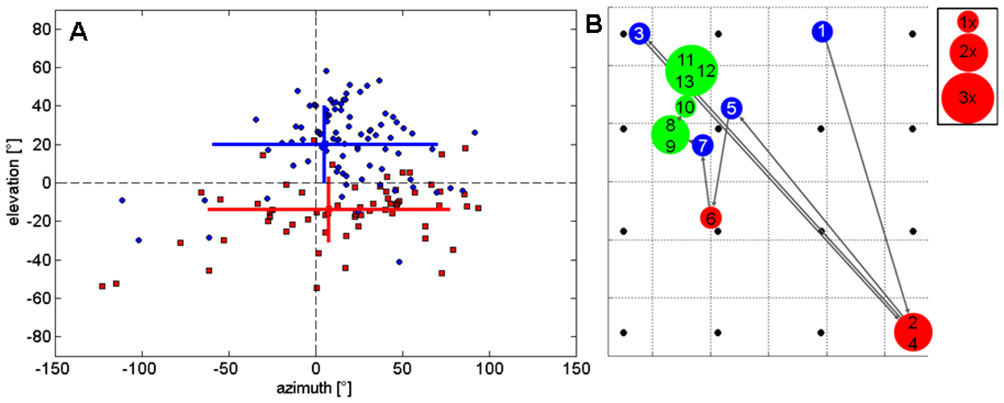
Separation of apparent beam directions of type 1 and type 2 signals. (A) Based on 19 sequences containing 66 type 1 signals (red) and 88 type 2 signals (blue) of bats flying towards the square array, measured angles between the flight path and the calculated apparent beam vector in the vertical (elevation) and horizontal (azimuth) projection plane are depicted. Each of the displayed elevation or azimuth angles corresponds to the angle between the flight direction and the apparent beam direction at the corresponding position of the bat (X) as marked in Fig 3A and 3C. The colored bars indicate the respective means and standard deviations. Note the clear separation between signal types. The vectors of type 1 signals were positioned mainly below and those of type 2 signals mainly above flight direction. (B) Pulse-to-pulse path of the calculated apparent (when at the array edges) or real (when within the array plane) beam maxima of an exemplary flight on the square array plane with successive numbers showing their order. Type 1 signals are depicted in red, type 2 signals in blue, and approach signals in green. Circles are proportional to the number of calls pointed to this location. The black dots depict the 16 microphones.

With the square array we measured an apparent mean vertical angle based on the apparent direction of 66 type 1 beams at -14° ± 17° (mean ± SD) relative to flight direction, whereas the mean vertical angle based on the apparent direction of 88 type 2 calls is 20° ± 20° (mean ± SD) relative to flight direction (Fig 4A). This resulted in an apparent mean vertical angular offset between the two search signals types of at least 33.9°. However, the geometric limits of the microphone array did not allow measuring larger angles. It is evident that real angles between the flight path and the calculated apparent beam vector exceed these apparent angles. The small offset of the mean to the right side is probably due to the angle in which the bats approached the array after leaving their roost. The pulse-to-pulse path of beam maxima on the array plane of one sequence is illustrated in Fig 4B. Type 1 calls clustered on the lower right edge of the array while type 2 calls were found towards the upper left edge. Approach calls stayed within the array limits and indicate that the bat was focusing on the obstacle during approach.

**Fig 5:**
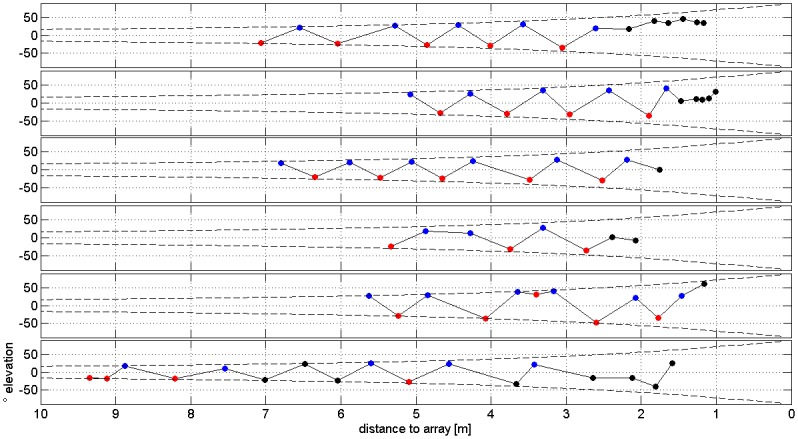
Separation of apparent beam directions of type 1, type 2, and approach signals. Six exemplary sequences of bats approaching the 6 m high chain array. The vertical beam direction is indicated by the vertical angle between the direction from the position of the bat (X) to the center of the microphone array (Y) and the apparent beam direction to the upper (Z) or lower end of the array as marked in Fig 3B. Differences in color indicate signal types (type 1 in red, type 2 in blue, approach calls in black). The dashed line shows the angle between the limits of the array to its center seen from the bats position and assuming the bat is centered in front of the array.

**Fig 6:**
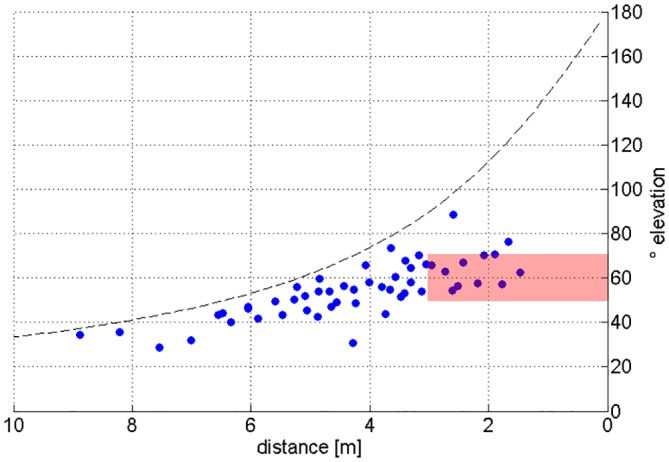
Apparent vertical angles between apparent call directions of type 1 and type 2 signals. The apparent vertical angle between the signal types was determined as the sum of the vertical angle for each call type as measured between the direction from the bat to the center of the 6 m high chain array and the direction to the calculated apparent beam maximum (see Fig 5). The dashed line depicts the maximum angle between the upper and lower border of the array as seen from the bats position under the assumption that the bat is centered in front of the array. The values between 4–10 m to the array are limited by this maximal possible angle value. The red rectangle below 3 m marks a range where array height did not limit the measured angles.

To alleviate the methodological limitation above, the 6 m high chain array was used to sample a larger vertical section of the bats’ detection space. Again many of the apparent beam directions either point on the lower or the upper array edge, particularly when the bat was at distance. As found previously, type 1 signals pointed downward and type 2 signals upward. Once bats closed in on the array, some calls were within the measurement limits (Fig 5). With the chain array, we found apparent vertical angles between the two call types of up to 88° (Fig 6). Starting at 3 m distance from the array, where array height did not limit the measured angles, the vertical angles between signal types ranged between 50–70°.

### Echolocation behavior based on head anatomy

**Fig 7:**
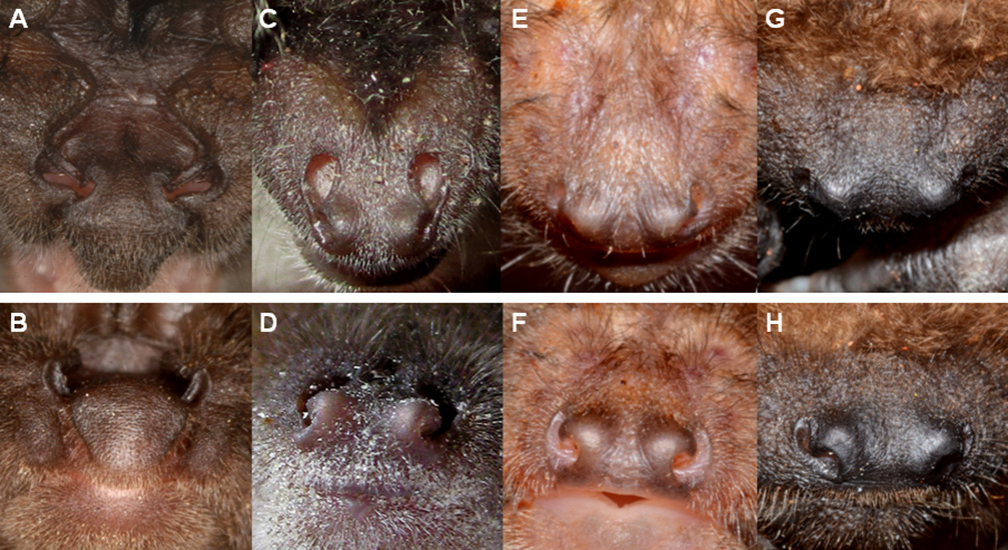
Nostril alignment in four species of Vespertilionid bats. Top view in the upper row and front view in the lower row of the noses of *Barbastella barbastellus* (A, B), *Plecotus auritus* (C, D), *Myotis bechsteinii* (E, F), and *Nyctalus noctula* (G, H). In *M*. *bechsteinii* and *N*. *noctula* the nostrils point forward and are not visible from above. In *B*. *barbastellus* and *P*. *auritus* the nostrils are turned upward and are therefore clearly visible from above and only partly visible in the front view. The front view photos were taken with the camera positioned in the plane of the upper jaw just in front of the bats and the top view photos were shot with the camera perpendicular to this plane positioned just above the bats.*Photo courtesy*: *(A*,*B) Christian Dietz; (C) Johanna Hurst; (D) Anna-Maria Seibert; (E-H) Laurent Arthur*.

**I**t was shown that barbastelle bats are able to emit echolocation calls through both their open mouth and nostrils [16,17]. In barbastelles, the mouth and nose openings point in different directions, with the nostril openings roughly perpendicular to the mouth opening. The nostrils are tilted upwards and open into a system of embedded lacunas leading all the way from the nose to the beginning of the ears, beyond and around the tragus. Comparing the external anatomy of the snout of several Vespertilionid species it is conspicuous that the embedded lacunas and upward pointing nostrils described for *B*. *barbastellus* are also found in the genus *Plecotus*, but not in other genera of this family whose nostrils open in a more forward direction (Fig 7) [18]. Lump-nosed bats (*Plecotus spec*.) are known to emit sounds through their nostrils as well [18]. Together with *Plecotus* species barbastelle bats belong to the tribe of Plecotini, and are therefore closer related to each other than to other species within the Vespertilionids.

## Discussion

Here we present new results on the SL of type 1 and type 2 signals and on the change of beam direction in *B*. *barbastellus* during search and approach flight. In summary, we found that the two types of search signals of *B*. *barbastellus* have nearly equivalent SL but are emitted in different directions along the vertical axis. The beams are separated by approximately 70° and the beam directions of type 1 signals are below flight direction whereas those of type 2 signals are above.

### Beam direction of approach calls

The apparent and real sonar beam directions of approach calls resemble those of type 2 calls, and largely point towards the upper area of the array (Figs 2–5). Even the recordings from a low and a high microphone of the array showed that the high microphone recorded not only type 2 signals but also approach signals with higher amplitude (Fig 1). The similarity in beam direction supports the conclusion that the broadband approach calls are derived from type 2 signals, as evidenced by sonagrams which show how type 2 calls morph into approach calls (Fig 1), [15,19].

### A hypothesis to explain the alternation between two emission directions

From single microphone recordings it was concluded that the amplitude variation between type 1 and type 2 signals indicate changes in beam direction in the vertical, and that they might be caused by up and down head movements [15]. In 15 high speed videos (by courtesy of Dietmar Nill) and on 39 photos of flying barbastelle bats (by courtesy of Laurent Arthur) we never found obvious changes in head position, which would indicate up down head movements. Another explanation for the observed change in emission direction could be that the upward directed type 2 signals are emitted through the upward pointing nose and downward directed type 1 signals through the mouth. This hypothesis is strongly supported by the observation that the type 1 signals are emitted through the mouth and the type 2 signals through the nose [16]. The possibility of switching between mouth and nose emission is supported by the finding that even if either the mouth or the nose of bats are plugged they are able to fly, avoid obstacles, and emit echolocation signals [17]. It was found that *B*. *barbastellus* and *Plecotus auritus* are able to emit signals either through the nose or through the mouth and that the oscillograms of the mouth and nose signals look rather similar [17]. It was also reported that *P*. *auritus* flies with mouth shut and that echolocation calls are emitted through the nostrils, which open upwards [18].

The switching between mouth and nose emission would explain the reported differences in SPL between call types in single microphone recordings only if the beams of the mouth and of the nose signals point in different directions. When looking at the head anatomy of *B*. *barbastellus* we found that the opening direction of the nostrils is nearly perpendicular to the direction of the mouth opening (Fig 7). If we assume that the pointing direction of nose and mouth opening determines the beam directions of the emitted signals we predict that the nose beam should be almost perpendicular to that of the mouth. This prediction corresponds well with our measurements that indicate a separation angle of about 70° in the vertical. We therefore conclude that *B*. *barbastellus* vary their emission direction in the vertical of about 70° by most likely emitting type 1 signals through the mouth and type 2 through the nose. The degree of angular separation between nose and mouth beam is determined by the anatomical relation between nostrils and mouth. This anatomical relation is fixed such that the angle between the two emission beams is likely invariable.

However, the emission directions of this fixed system are additionally dependent on the aiming of the head. We found that the beam directions of most type 1 calls were below flight direction whereas those of most type 2 calls were above. This implies that in horizontal flight the mouth opening points downward and the nostrils point upward.

*Pipistrellus pipistrellus*, a species that emits signals only through the mouth, is able to move its head and with it the beam direction [20]. We therefore assume that *B*. *barbastellus* also has the ability to move its head around a roll, pitch and yaw axis and with it the beams of the anatomically fixed nose and mouth emitters. Some of our data indicate that these bats also make head movements. Between succeeding signals beam direction was not only changed in the vertical but also tilted to the side around a roll axis which is indicated by the measured horizontal angles in Fig 4A. The scan path of an example flight in Fig 4B also shows that the bat was capable of turning its head such that the beam moved from right/down to left/up and back. Another hint of head movements around the pitch axis comes from directional changes of the approach signals in Figs 4 and 5. The direction of these nose signals moves from the upper end of the array more to the center indicating that the bats fixate a target with the nose beam. We predict that such a fixation with the nose beam will also occur in bats approaching an insect.

### Adaptive value of emitting two types of search calls with similar SL in different directions

*B*. *barbastellus* forages near vegetation during aerial-hawking and preys mainly on tympanate moths [3,4,12]. According to this foraging behavior, barbastelles are attributed to the guild of “edge space aerial-hawking foragers” that search for prey near background targets [6]. Foraging and echolocation behavior of bats have been adapted to the task they have to perform while searching and acquiring food. All edge space foragers share similar adaptations, and their echolocation systems display many similarities. However, this does not exclude species-specific differences that reflect niche partitioning within guilds [6]. If we compare the echolocation behavior of *B*. *barbastellus* with that of other edge space foragers we find many similarities, but also some distinct differences which may account for niche differentiation.

The signal pattern of foraging *B*. *barbastellus* generated only by type 2 search signals and approach signals is rather similar to that of other edge space aerial-hawking foragers [15,19]. The bats emit shallowly downward modulated search signals of moderate bandwidth (frequency range of 45–32 kHz). The signals are varied in duration (around 3–8 ms), most likely in relation to the distance to the background. The initial more shallow modulated part of the signal improves detection, and the steeper modulated terminal part improves localization of prey [21]. Sometimes the bats skip a sound emission which is indicated by longer intervals. After detection of prey with a long type 2 signal the bats switch to broadband approach signals (frequency range of 52–23 kHz). The first signal of an approach may be even longer than the preceding type 2 signal (transition signal according to [19]) but afterwards signal duration and pulse interval are reduced in the typical way as in other aerial-hawking species approaching prey. The approach ends with a typical buzz which is indicated by pulse intervals below 8 ms. Often the buzz takes quite long and is interrupted by one or several longer pulse intervals (see Fig 3 in [15]). The prolonged buzz with much more signals than in a short buzz most likely indicates that the bats pursue prey which try to escape. In summary, in foraging *B*. *barbastellus*, the pattern of only type 2 and approach signals is rather similar to that of other edge space aerial-hawking foragers.

However, we also found distinct differences when comparing the echolocation behavior of *B*. *barbastellus* with that of other edge space aerial-hawking foragers. One big difference is that the type 2 and the approach signals are most likely emitted through the nose. The emission of signals through the nose is also found in bats of the genus *Plecotus* which—together with the barbastelles—belong to the tribe Plecotini. This phylogenetic relationship probably indicates that the two genera had a common ancestor with nose emission [22]. It is striking that the nostril alignment of those two genera differs from the other Vespertilionids in that it contains more cavities with nose openings turned upwards (see also Fig 7) [3,18].

But why are other genera within the family of Vespertilionidae able to forage with mouth-bound signals alone while barbastelle bats have evolved two different emitters and use two different signal types with equal SLs (81–82 dB SPL rms re 1 m) far below those of other aerial-hawking foragers that use SLs of 101–114 dB SPL rms re 1 m [23, 24]? This rather low SL has been interpreted as an adaptation that allows *B*. *barbastellus* to hunt successfully for tympanate moths. Many moths can hear and react with escape responses as soon as they detect an approaching bat [2,25,26]. Most edge space aerial-hawking foragers have high SLs which allow for long detection distances. However, these high SLs have the disadvantage that moths can detect the bats early enough to initiate often-successful escape maneuvers. Goerlitz et al. (2010) [4] were the first to propose that *B*. *barbastellus* might use some kind of “stealth echolocation” and produces search signals with low SLs that are inconspicuous to eared moths. They determined a SL for type 1 signals of only 94 dB peSPL re 0.1 m which corresponds to 74 dB peSPL re 1 m. We assume that this value is too low as their method probably delivered weaker apparent beam maxima instead of louder real beam maxima. The main peak in the histogram of their SPLs (Fig 3B in [4]) has an upper limit near 104 dB peSPL re 0.1 m which corresponds to 84 dB peSPL re 1 m. It is difficult to compare the peSPL measurements of [4] with our SPL rms measurements. Their peSPL values are related to the rms value of a sine wave with a constant amplitude whereas the SPL rms measurements of our sonarbeam software uses the time-varying call amplitude. The two values would be the same if the bats kept a constant peak amplitude over the entire signal. In barbastelles the call amplitude does not change very much within the signal (see Fig 1 in [15]). According to the envelope of barbastelle signals we estimate that our SPL rms value is between 1–4 dB smaller than the peSPL values of [4]. The subtraction of an estimated 3 dB from the value taken from the histogram in Fig 3B in [4] results in a SL of 81 dB SPL rms re 1 m which corresponds very well to the average SL of the type 1 signals of our measurements.

For *B*. *barbastellus*, foraging for large moths with a target strength of -36 dB (re 1 m) the detection distance is about 3 m if we assume a best frequency of 40 kHz, a detection threshold of 20 dB, a temperature of 15°Celsius, and a humidity of 50% (all calculations according to [27]). Under similar conditions, *N*. *leisleri* could detect this prey over a distance of 8.5 m if we assume a SL of 107 dB SPL rms re 1 m [27] and a best frequency of 28 kHz. The neural maximum for echolocation detection distances of the moth *Noctua pronuba* by foraging *B*. *barbastellus* and *N*. *leisleri* was measured in the field [4]. The more sensitive A1 neuron of the moth always reacted to the search signals of *N*. *leisleri* before the bat heard the moth echoes. The moth’s A1 detection distance was with 33 m far beyond the 9 m detection distance of the bat calculated from our data. This early warning gives eared moths the possibility to start with escape movements in time. In foraging *B*. *barbastellus* the moth’s A1 neuron detection distance of 4 m was close to the bat’s detection distance of 3 m which we calculated from our data. This suggests that eared moths have far less chance to escape barbastelles by evasive movements. Therefore, our findings lend support to Goerlitz et al. (2010) [4] who stated “*B*. *barbastellus* uses a stealth echolocation strategy by emitting low-amplitude calls, a strategy previously suggested by Fenton & Fullard (1979) [28] and by Surlykke (1988) [29] and now supported with field-based measurements”.

In contrast to [4] we do not assume that *B*. *barbastellus* use type 1 signals as search signals for prey. We hypothesize that these signals have evolved for a different function. The SLs of the type 1 and type 2 signals of *B*. *barbastellus* are 20–25 dB lower than the SLs of other edge space aerial-hawking foragers. This has the advantage of allowing *B*. *barbastellus* to approach very close to eared moths without provoking evasive movements, but at the cost of a highly reduced detection distance for prey and background targets. When comparing the calculated maximum detection distances for prey of type 2 signals with those of search signals of *N*. *leisleri* [27] we found a lower detection distance by about 1/3^rd^ in type 2 signals (which subsequently reduces the search volume to about 1/27^th^). Additionally, the detection distance for vegetation in the flight path is strongly reduced from 23 m in *N*. *leisleri* to 8 m in type 2 signals of *B*. *Barbastellus* (see [27]).

In *B*. *barbastellus* foraging above a forest canopy with type 2 signals pointing slightly upward, the detection and the evaluation of canopy echoes are hampered as the directionality of signal emission and also of echo reception substantially reduces the SPL of type 2 echoes from below. If we assume that the perceived SPLs of echoes returning at an angle of 45–60° relative to the beam’s emission direction are at least attenuated by 20–30 dB (estimated according to [30,31]) the detection distance for forest at an downward angle of 45–60° relative to the beam direction would be less than 1–2 m. For echoes returning vertically from below the detection distances would be even smaller. These short detection distances would make it difficult for the bat to forage above vegetation with type 2 signals alone. However, the emission of type 1 signals downward improves the chance to detect and evaluate forest echoes from below. With a type 1 signal that is emitted with a downward beam direction of 45° the detection distance for a forest in beam direction is at about 9 m [27]. This is comparable to a *N*. *leisleri*, which can detect forest echoes returning from an angle of 45° below the horizontal emission direction up to a distance of 12 m (assuming a reasonable 20 dB attenuation of the perceived echo SPL). In barbastelles which emit type 1 signals at a downward emission angle of 45° even the echoes returning vertically from below would allow detection distances of up to 2 m for forests, 5 m for meadows, and 10 m for water surfaces (again assuming a reasonable 20 dB attenuation of the perceived echo SPL at an angle of 45°relative to the beam direction). We therefore conclude that the downward directed type 1 signals have evolved to determine the bat’s position in relation to the environment below. Such a strategy has previously been suggested [15]. With downward directed type 1 signals *B*. *barbastellus* overcomes the disadvantage of the stealth strategy with the rather short detection distances for the environment below the bat due to low emission SLs of the more upward directed type 2 signals. This strategy does not exclude that in favorable situations echoes of type 1 signals are also able to locate prey such that the overall search volume can be increased. However, the observation that type 1 signals are always rather stereotyped and, in contrast to type 2 signals, do not change according to foraging situation [15] suggests that they are not primarily used as search signals for prey.

A different argument to explain why *B*. *barbastellus* evolved two types of echolocation signals was used by Barataud (2004) [19]. He suggested that bats use two signal types which differ in intensity, structure, and frequency to deceive tympanate moths by mimicking the presence of two bats at different distances with sufficiently low repetition rates so as to not provoke the prey’s escape behavior. However, [4] and the present study suggest that *B*. *barbastellus* actually use a stealth strategy to improve their hunting success. Given that both signal types have low SLs and cannot be heard by the moths, it is unlikely that the two signal types have evolved to deceive prey.

In conclusion, we found that foraging *B*. *barbastellus* emit two signal types of equally low SL in different directions. In relation to flight direction the beams of type 2 signals point upward, and that of type 1 signals downward. The beams are separated by a fixed vertical angle of approximately 70°. Barbastelle bats are able to emit signals through the mouth or the nose and these signals occur at roughly perpendicular angles to one another. This suggests that type 1 signals are most likely emitted through the mouth and type 2 through the nose. In addition, this fixed double emission system can be actively adjusted up or down around the pitch axis, and tilted around the roll axis of the head. We hypothesize that the “stealth” echolocation system of *B*. *barbastellus*, with two different signal types of low SL which are emitted in different emission directions, is bifunctional. The upward directed nose signals are used for the search of, and the approach to, prey. Their low SL prevents an early detection of bats by eared moths at the expense of a significantly reduced detection range for the environment below the bat. The more downward directed mouth signals have evolved to compensate for this disadvantage. These second signals are largely used for spatial orientation and biotope recognition.

## Materials and Methods

### Ethics Statement

No specific permits were required for the described field studies since only sound recordings were made and no specimens were sampled and/or handled. No specific permits were required for the locations where recordings took place. Private land was accessed with the permit of Laurent Arthur from the Muséum d’Histoire Naturelle de Bourges, France. Field studies did not disturb endangered or protected species.

### Animals and recording sites

Barbastelle bats (*Barbastella barbastellus*) were recorded at three locations with known roosts (referred to as location # 1–3) in Central France near Bourges from June 29 –July 20, 2009, and June 3 –June 5, 2010, between 22:00 and 23:00 hours (MEZ). The bats usually left their roost at approximately 22:00 hours. Before we set up the recording equipment we determined the common flight route of bats emerging from the roost. The arrays were positioned perpendicular to the main flight corridor with a distance of 6–35 m from the roost to ensure that the bats flew more or less centered on and straight towards the array. Bats approached the array at heights of 2–6 m.

Pseudoreplication by recording an individual several times cannot be completely excluded since recordings were made on consecutive nights. However, bats were leaving their roost and we assume that per night any given animal was recorded only once with approximately the same number of alternating call types.

### Experimental setup

We used nearly omnidirectional Knowles (FG-23329, Itasca, IL, USA) microphones with known angular sensitivity at different frequencies fixed in small custom-made housings. Recordings were made using two different array-configurations. Most recordings were made with a **square microphone array** with 16 microphones arranged on nylon strings (ø 0.7 mm) in a 4x4 vertical planar grid attached to an aluminum frame (4x4 m). The microphones were equally spaced with 0.80 m apart on the horizontal and vertical axis facing the bats (Fig 1B). With the square array the lowest microphone row was elevated approx. 1.5–2.0 m above ground.

At one location we additionally used a setup with three 6-m-long microphone chains which were kept in a vertical position by two flag poles. At the **chain array** the microphones were attached to nylon strings in a 4-8-4 grid with a vertical spacing of 2.0, 0.85, and 2.0 m, respectively. The nylon strings were spaced with 0.80 m on the horizontal axis (Fig 3E). In that setting, the lowest microphone row was approx. 0.3 m above ground.

The recorded signals of each of the 16 microphones were amplified using a custom-made amplifier and digitized by two 8-channel National Instruments (NI-PXI 6123) cards at 500 kHz and 16 bit sampling rate and fed into a ring buffer using custom-made software (SIMI-MOTION version 7.5.0.288). After stopping the recording, the last four seconds in the buffer were stored on a laptop computer as Waveform Audio (.wav) files.

### Database

24 sequences of approaching barbastelle bats recorded with the square array were analyzed; these contained 337 calls in total. One sequence originated from location #1, three sequences from location #2, and the remaining 20 sequences were recorded at location #3. These calls were all emitted at distances of 1–10 m from the microphone array. We recorded 86 type 1, 110 type 2, and 141 approach calls. With the chain array, six sequences containing 26 type 1 calls, 30 type 2 calls, and 28 approach calls from location #3 were analyzed. Sequences were chosen based on good signal quality (good signal-to-noise ratio at all 16 receivers) and favorable flight path towards the microphone array.

### Flight path reconstruction

Each bat’s flight path was reconstructed using a custom-made Matlab (Mathworks, Natick, MA, USA) script to calculate the position of the bat at signal emission by using the time of arrival differences (TOADs) between microphones. The TOADs between the upper left array microphone and each of the other 15 microphones were computed by cross correlating the same echolocation call. The position of the sound source was then computed using least-squares approximation [32]. In preliminary tests with a stationary ultrasonic speaker emitting a bat-like 10 ms long FM sweep from 80–10 kHz at different known positions in front of the array we found that the positioning error in all three dimensions was no more than 2–3% of the distance to the array.

### Signal analysis

Several signal parameters, including call duration, pulse intervals, bandwidth, peak frequency, and start and terminal frequency were visually inspected and measured in color spectrograms (FFT 512, Hann window, dynamic range of 90 dB) using custom-made software (Selena, University of Tübingen, Germany). Due to auto-padding and time interpolation, the spectrograms were plotted with a temporal resolution of 0.05 ms and a spectral resolution of 215 Hz. The beginning and end of signals were measured in the spectrograms using the automatically applied criterion of -6 dB below highest amplitude. To avoid pseudoreplication in the calculation of mean signal parameters, only the mean of each sequence was determined and used for subsequent analyses (Table 1).

### Sonar footprint and sonar beam

The TOAD positions along with the corresponding time stamps for each signal were used as input by a Matlab-based software (Sonarbeam [33]), to calculate sonar footprints on the array plane and polar graphs of the sonar beams from the bat’s perspective with color-coded SPLs (Fig 2). Geometrical spreading loss, atmospheric attenuation, and the individual microphone angular sensitivity were accounted for in a frequency-dependent manner. The reconstructed beam maxima (maximum value in the polar graph of a beam) are referred to as real maxima, when beam maximum values fell within the array, or as apparent maxima, when the maximum values were positioned at the border of the array. Preliminary tests with an artificial sound source indicated that the accuracy of beam reconstruction was sufficient to determine changes in angular orientation of the sonar beam. Position errors for the beam maximum of approximately 2° were measured [34].

### Determining calling direction

For each reconstructed beam recorded with the square or chain array, the direction of the real or apparent beam maximum was determined and displayed as a vector on the flight path color-coded depending on call types (type 1 calls in red, type 2 calls in blue, approach calls in black; Fig 3). The real or apparent beam direction in relation to the flight path was also described by the azimuth and elevation angle of the real or apparent beam direction in relation to flight direction (Fig 4A). Additionally, changes in beam direction were indicated by the path of the real and apparent beam maxima of succeeding pulses on the microphone grid (Fig 4B).

To describe changes of beam direction in recordings with the 6 m high chain array, we determined the horizontal and vertical angle between the center of the array and the reconstructed real or apparent beam maximum for succeeding pulses. However, the spatial resolution in the horizontal was too small for a precise beam reconstruction. Therefore we present only data describing the vertical deviation from the center of the array (Fig 5). In most recordings with the chain array, the bat passed the array on either side. The array therefore only recorded narrow vertical sections of the beams lateral to the beam axis so that our results describe only changes of apparent beam directions in the vertical plane.

### Source level determination

For the calculations of the SLs of both call types, the frequency range was limited to 20–60 kHz by bandpass filtering, and sound pressure levels are given in dB SPL rms re 20 μPa at 1 m. Only 14 of the recorded type 1 calls and 10 of the type 2 calls were centered within the array plane in such a way as to ensure that only real beam maxima (and thus absolute SLs), and not apparent beam maxima, were measured. However, the apparent source levels (ASLs) and the absolute SLs did not differ significantly in type 2 signals and only slightly in type 1 signals (for type 1 *t* = 2.13, *p* = 0.04; for type 2 *t* = -0.02, *p* = 0.98). These data suggest that the real beam maxima outside of the array must have been rather close to the apparent beam maxima at the edge of the array.

### Methodological limits

We investigated the SL and the variation of beam direction with arrays of 16 microphones. This approach had the advantage that a substantial part of the acoustic beam could be sampled. The sampled part of the beam increased as the bat closed in on the array. When the maximal intensity of the beam was within the array, we could determine the SL of the signals and the exact beam emission direction. When the beam maximum was not within the array, the apparent beam maximum and the apparent beam direction still provided information on the ASL and on changes of beam direction between succeeding signals. We are aware that the array design also posed certain limits. The square array (4x4 microphones) was too small in the vertical to record centered beam maxima in a sequence of succeeding type 1 and type 2 signals. Either the bats were too far away, and thus the beams pointed above and below the array, or they were so close that bats switched to approach calls. The chain array (4+8+4 microphones) had sufficient vertical but poor horizontal spatial resolution. However, apparent beam maxima of succeeding pulses gave some indications about the directional changes of the beam. They also indicated whether the beam direction was above or below flight direction (Fig 5).

### Statistics

Statistical analysis was performed in JMP (SAS Institute Inc., Cary, NC, USA). To test for the differences between the two signal types, a student’s t-test was performed using standard significance criteria (*p* ≤0.05). To avoid pseudoreplication when calculating the mean signal parameters from sequences containing a different number of signals, only the mean of each sequence was determined and used for further analyses.
